# Subdiffuse scattering model for single fiber reflectance spectroscopy

**DOI:** 10.1117/1.JBO.25.1.015001

**Published:** 2020-01-09

**Authors:** Anouk L. Post, Henricus J. C. M. Sterenborg, Fransien G. Woltjer, Ton G. van Leeuwen, Dirk J. Faber

**Affiliations:** a Amsterdam UMC, University of Amsterdam, Cancer Center Amsterdam, Amsterdam Cardiovascular Sciences, Department of Biomedical Engineering and Physics, Amsterdam, The Netherlands; b The Netherlands Cancer Institute, Department of Surgery, Amsterdam, The Netherlands

**Keywords:** subdiffuse scattering, backscattering, reflectance spectroscopy, optical properties, single fiber reflectance spectroscopy

## Abstract

To detect small-scale changes in tissue with optical techniques, small sampling volumes are required. Single fiber reflectance (SFR) spectroscopy has a sampling depth of a few hundred micrometers. SFR spectroscopy uses a single fiber to emit and collect light. The only available model to determine optical properties with SFR spectroscopy was derived for tissues with modified Henyey–Greenstein phase functions. Previously, we demonstrated that this model is inadequate for other tissue phase functions. We develop a model to relate SFR measurements to scattering properties for a range of phase functions, in the absence of absorption. Since the source and detector overlap, the reflectance cannot be accurately described by diffusion theory alone: SFR measurements are subdiffuse. Therefore, we describe the reflectance as a combination of a diffuse and a semiballistic component. We use the model of Farrell et al. for the diffuse component, solved for an overlapping source and detector fiber. For the semiballistic component, we derive a new parameter, $p_{\mathrm{sb}}$, which incorporates the integrals of the phase function over 1 deg in the backward direction and 23 deg in the forward direction. Our model predicts the reflectance with a median error of 2.1%, compared to 9.0% for the currently available model.

## 1 Introduction

Reflectance spectroscopy techniques are used to relate tissue optical properties to various types of diseases. Depending on the clinical question, an important consideration in choosing a spectroscopic technique is its sampling volume. Since optical properties are averaged over this volume, techniques with small sampling volumes are required to detect local, small-scale changes in tissue. Superficial sampling is relevant for early detection of cancer in general, and especially for the detection of (premalignant) lesions in the epithelium of, e.g., the esophagus or the colon, since the epithelium is only a few hundred micrometers thick. Single fiber reflectance (SFR) spectroscopy is a technique with a sampling depth in the order of a few hundred micrometers and has been used in a number of studies in the field of oncology.^1^^–^^6^ SFR spectroscopy uses a single fiber, in contact with tissue, to emit and collect light, connected to a broadband light source and a spectrograph to detect the steady-state reflectance versus wavelength. From the measured spectra, tissue optical properties can be derived and related to the disease state of tissue.

Reflectance in terms of optical properties can be expressed in general as

$$R[\mu_{s},p(\theta ),\mu_{a}]=\overset{\infty }{\underset{0}{\int }}p[l;\mu_{s},p(\theta )]e^{-\mu_{a}l}dl,$$ (1)

where $\mu_{s}$ is the scattering coefficient, p(θ) is the phase function (the probability distribution of scattering angles), and $\mu_{a}$ is the absorption coefficient. Equation (1) describes the reflectance as the sum of photons with different pathlengths, according to the pathlength distribution p(l). The pathlength distribution only depends on the scattering properties, $\mu_{s}$ and p(θ). Absorption is accounted for by weighting each pathlength contribution according to the Beer–Lambert law (the exponential term in the integral). Equation (1) enables separate modeling of the effects of scattering and absorption on reflectance.

Currently, a single model is available to derive optical properties from SFR measurements, developed by Kanick et al.^7^^,^^8^ This model consists of two parts: one part describes reflectance as a function of tissue-scattering properties in the absence of absorption ($R_{0}$)^7^ and the second part includes absorption.^8^ Kanick et al. simplified the modeling by approximating Eq. (1) by Eq. (2), describing reflectance as reflectance in the absence of absorption ($R_{0}$) multiplied by a factor to account for absorption, featuring $\mu_{a}$ and an effective pathlength ⟨L⟩:

$$R=R_{0}e^{-\mu_{a}\langle L\rangle }.$$ (2)

The model for $R_{0}$ describes the reflectance as a function of the product of the reduced scattering coefficient and the fiber diameter ($\mu_{s}^{\prime }d$), the phase function parameter γ (which depends on the first and second Legendre moments of the phase function), the fiber numerical aperture (NA) and the refractive index of the sample ($n_{\text{sample}}$):

$$R_{0}={\left(\frac{\mathrm{NA}}{n_{\text{sample}}}\right)}^{2}\cdot (1+0.632\gamma^{2}e^{-2.308\gamma^{2}\cdot \mu_{s}^{\prime }d})\cdot [\frac{{\left(\mu_{s}^{\prime }d\right)}^{0.574\gamma }}{2.308\gamma^{2}+{\left(\mu_{s}^{\prime }d\right)}^{0.574\gamma }}].$$ (3)

This model was derived using Monte Carlo (MC) simulations of tissues with modified Henyey–Greenstein (MHG) phase functions. We previously demonstrated that Eq. (3) does not predict the reflectance well for tissues with phase functions other than MHG.^9^

For many tissue types, it has been shown that scattering is more backward directed than can be described by the MHG. The two-term Henyey–Greenstein (TTHG) has been shown to describe the phase function of tissue in, e.g., the liver,^10^^,^^11^ uterus,^10^ brain,^12^ breast,^13^ and muscle,^14^ and for blood, it has been shown that the phase function is better represented by the Reynolds–McCormick (RMC, also known as Gegenbauer) phase function.^15^^,^^16^ The phase function of a specific tissue under investigation is generally not known. For example, even though a TTHG phase function has been measured in the breast, this does not imply that breast tissue will always have a TTHG phase function. The phase function will depend on the type of breast tissue that is investigated (e.g., fat or ducts) and it might change during progression from healthy to diseased tissue. Therefore, to ensure an accurate determination of the optical properties from SFR measurements, a model that is valid for the wide range of phase functions that can be encountered in tissue is essential.

In this paper, as a first step toward a comprehensive SFR spectroscopy model that includes both scattering and absorption properties, we develop a model that accurately relates the reflectance in the absence of absorption ($R_{0}$) to tissue-scattering properties for a wide range of tissue phase functions. This model can be expanded in the future to also include absorption. We model $R_{0}$ as a combination of a diffuse and a semiballistic component. For SFR spectroscopy, diffusion theory alone is not appropriate to model reflectance as a function of tissue optical properties. Since a single fiber is both the source and detector, the distance between the location where photons enter the tissue and where they are detected is generally less than the transport mean free path $1/\mu_{s}^{\prime }$. SFR measurements are, therefore, in the so-called subdiffuse regime, where the measured reflectance is a combination of detected photons that underwent a large number of scattering events (diffuse photons) and detected photons that underwent only a few scattering events (semiballistic photons). We consider the detected photons semiballistic when they have experienced a single backscattering event in combination with an arbitrary number of forward-scattering events.

We describe the diffuse component using the model of Farrell et al.^17^ for spatially resolved diffuse reflectance, as solved by Faber et al. for an overlapping source and detector fiber.^18^ The diffuse component of the reflectance depends only on the product $\mu_{s}^{\prime }d$, where $\mu_{s}^{\prime }=\mu_{s}(1-g_{1})$, $\mu_{s}$ is the scattering coefficient and $g_{1}$ is the scattering anisotropy (first Legendre moment of the phase function). After a few scattering events, photon direction is randomized. Therefore, diffuse reflectance does not depend on the details of the tissue phase function but only on the scattering anisotropy $g_{1}$. For semiballistic photons, the photon direction is not fully randomized and, thus, measurements are more sensitive to the shape of the phase function.^19^^,^^20^ Therefore, we searched for a parameter that would optimally capture the phase function influence on the semiballistic contribution to the reflectance. We developed the parameter $p_{\mathrm{sb}}$ and showed that it improves the prediction of the semiballistic contribution to the reflectance compared to other parameters that have been proposed to model subdiffuse reflectance (γ,^21^ δ,^22^ σ,^23^ and $R_{p\mathrm{NA}}$^9^). Therefore, we model the semiballistic contribution to the reflectance as a function of $\mu_{s}^{\prime }d$ and $p_{\mathrm{sb}}$. Based on MC simulations with MHG, TTHG, and RMC phase functions, we show that our model (combining the diffuse and semiballistic contributions) predicts the reflectance more accurately, compared to the currently used model from Kanick et al.^7^ for a range of phase functions, $\mu_{s}^{\prime }d$ values and NAs.

## 2 Methods

### 2.1 Monte Carlo Simulations

We performed absorption-free MC simulations to investigate the relationship between $R_{0}$ and tissue-scattering properties for SFR spectroscopy. We modified^9^ the software of Prahl et al.^24^ and Wang et al.^25^ to enable simulation of an overlapping source/detector geometry and to allow the use of any phase function using the method of Zijp and ten Bosch.^26^ Photons were launched from a location based on a uniform distribution across the source with an angle from a uniform angular distribution within the launch/acceptance angle of the fiber $\theta_{\mathrm{acc}}$, where $\theta_{\mathrm{acc}}=\arcsin(\mathrm{NA}/n_{\text{sample}})$. Photons were detected if they reached the fiber face at an angle within $\theta_{\mathrm{acc}}$. We performed simulations for NAs of 0.10, 0.22, and 0.50. The refractive index was set to 1.35 for the tissue, 1.45 for the fiber face, and 1.00 for the medium above the sample. For all the MC simulations, we ran each simulation three times and ensured that enough photons had been launched such that the standard deviation over the mean of the reflectance for each set of three simulations was less than 2%. In conventional MC simulations that include absorption, the photon weight is reduced at the end of every step. There, to prevent endless tracking of photons with small weights, usually a “roulette” routine is implemented to terminate these photons when their weight drops below a certain value. In our absorption-free MC simulations, weights are maintained and another process for termination is needed. We terminated photons at a set distance from the fiber face (termination distance) and we checked that at least 99.9% of detected photons had traveled up to a distance from the fiber face less than 75% of the termination distance.

We performed simulations using MHG, TTHG, and RMC phase functions, employing the parameters specified in Table 1 and applying the restrictions $g_{1}\geq 0.5$ and $g_{2}<0.9$ to exclude biologically unreasonable phase functions. This resulted in 207 phase functions (15 MHG, 146 TTHG, and 46 RMC) with fairly equally distributed $g_{1}$ values between 0.5 and 0.94. For each phase function, we performed simulations for four values of $\mu_{s}^{\prime }d$: 0.1 ($\mu_{s}^{\prime }=10\;{\mathrm{cm}}^{-1}$, d=100  μm), 1 ($\mu_{s}^{\prime }=100\;{\mathrm{cm}}^{-1}$, d=100  μm), 5 ($\mu_{s}^{\prime }=500\;{\mathrm{cm}}^{-1}$, d=100  μm), and 9 ($\mu_{s}^{\prime }=100\;{\mathrm{cm}}^{-1}$, d=900  μm).

**Table 1:** Parameters employed in the selection of phase functions.

Phase function	Parameters
MHG	$0.01\leq g_{\mathrm{HG}}\leq 0.95$, 10 linear steps
	0.01≤α≤0.99, 10 linear steps
TTHG	0.5≤α≤0.9, 3 linear steps
	0.91≤α≤0.99, 5 linear steps
	$0.05\leq g_{f}\leq 0.95$, 10 linear steps
	$-0.95\leq g_{b}\leq -0.05$, 5 linear steps
RMC	0.01≤α≤2.5, 10 linear steps
	$0.01\leq g_{R}\leq 0.95-0.2\cdot \alpha$, 10 linear steps

The simulations with $\mu_{s}^{\prime }d=0.1$ were used to derive a new parameter to describe the semiballistic contribution to the reflectance $p_{\mathrm{sb}}$. To confirm that, for $\mu_{s}^{\prime }d=0.1$, the detected photons are indeed semiballistic, we determined the fraction of detected photons that underwent a single backscatter event in combination with an arbitrary number of forward-scattering events. The simulations with $\mu_{s}^{\prime }d=0.1$ were used to investigate the relationship between $R_{0}$ and $p_{\mathrm{sb}}$, γ, δ, σ, and $R_{p\mathrm{NA}}$.

In addition, we performed simulations with 10 phase functions (selected such that they yielded reflectance values between $10^{-5}$ and $4\cdot 10^{-3}$ in 10 equal steps on a logarithmic scale, for an NA of 0.22 and $\mu_{s}^{\prime }d$ of 0.1) for 20 values of $\mu_{s}^{\prime }d$ between 0.1 and 100 ($\mu_{s}^{\prime }=10$ to $10000\;{\mathrm{cm}}^{-1}$, equally spaced in 20 steps on a logarithmic scale, d=100  μm). All the simulations combined were used to derive constants in our scattering model, as well as to test the accuracy of this new model.

We also investigated the limits of the new model for low values of $\mu_{s}^{\prime }d$. Since part of our model is based on diffusion theory, it can be expected that there is a limit where $\mu_{s}^{\prime }d$ is too low for diffusion theory to accurately describe part of the reflectance. Therefore, we performed MC simulations for the same 10 phase functions, with $\mu_{s}^{\prime }d$ values of 0.005, and 0.01 to 0.09 in steps of 0.01 (d=100  μm), and we compared the simulated reflectance to the modeled reflectance.

### 2.2 Scattering Model

We model the reflectance ($R_{0}$) as the sum of a diffuse ($R_{\mathrm{SFR},\mathrm{dif}}$) reflectance and a semiballistic ($R_{\mathrm{SFR},\mathrm{sb}}$) reflectance:

$$R_{0}=R_{\mathrm{SFR},\mathrm{dif}}+R_{\mathrm{SFR},\mathrm{sb}.}$$ (4)

The ratio between the contribution of semiballistic and diffuse photons will depend on the scattering properties of the tissue. For example, for high values of $\mu_{s}^{\prime }d$, almost all detected photons will be diffuse, whereas for low values of $\mu_{s}^{\prime }d$, most detected photons will be semiballistic. Therefore, we rewrite Eq. (4) using this ratio

$$X=\frac{R_{\mathrm{SFR},\mathrm{sb}}}{R_{\mathrm{SFR},\mathrm{dif}}}$$ (5)

as

$$R_{0}=(1+X)\cdot R_{\mathrm{SFR},\mathrm{dif}}.$$ (6)

#### 2.2.1 Diffuse component

We describe the diffuse component, $R_{\mathrm{dif}}$, using the model of Farrell et al.^17^ for spatially resolved reflectance as solved by Faber et al.^18^ for an overlapping source and detection fiber in the absence of absorption:

$$R_{\mathrm{dif}}(\mu_{s}^{\prime }d)=\frac{\pi }{4}\cdot d^{2}\cdot \overset{d}{\underset{0}{\int }}R_{\mu a=0}(\rho ,\mu_{s}^{\prime })\cdot p(\rho ,d)d\rho .$$ (7)

Here, $R_{\mu a=0}(\rho ,\mu_{s}^{\prime })$ is the diffuse reflectance versus radial distance for a pencil beam illumination,

$$R_{\mu a=0}(\rho ,\mu_{s}^{\prime })=\frac{1}{4\pi }\{\frac{{\mu_{s}^{\prime }}^{2}}{{\left[1+{\left(\mu_{s}^{\prime }\cdot \rho \right)}^{2}\right]}^{\frac{3}{2}}}+\frac{(1+\frac{4A}{3})\cdot {\mu_{s}^{\prime }}^{2}}{{\left[{\left(1+\frac{4A}{3}\right)}^{2}+{\left(\mu_{s}^{\prime }\cdot \rho \right)}^{2}\right]}^{\frac{3}{2}}}\}$$ (8)

and p(ρ,d) is the distribution function of distances over the fiber face,

$$p(\rho ,d)=\frac{16\rho }{\pi d^{2}}\;\cos^{-1}(\frac{\rho }{d})-\frac{16}{\pi d}{\left(\frac{\rho }{d}\right)}^{2}\sqrt{1-{\left(\frac{\rho }{d}\right)}^{2}.}$$ (9)

Equation (9) describes the distribution of distances between two randomly placed points on a disk with diameter d, which is a classic problem in the field of geometric probability.^27^ The parameter A depends on the refractive index mismatch between the tissue and the fiber.^28^ For a tissue refractive index of 1.35 and a fiber refractive index of 1.45, A is equal to 1.027.

Using Eqs. (7)–(9), $R_{\mathrm{dif}}$ models the fraction of photons that are diffuse and arrive at the fiber face. Photons are only detected if they arrive at the fiber at an angle smaller than or equal to the acceptance angle of the fiber. To account for the acceptance angle of the fiber, we need to multiply $R_{\mathrm{dif}}$ by the collection efficiency of the fiber ($\eta_{c}$):

$$R_{\mathrm{SFR},\mathrm{dif}}=\eta_{c}\cdot R_{\mathrm{dif}}(\mu_{s}^{\prime }d).$$ (10)

The collection efficiency of the fiber depends on the angular distribution of photons reaching the fiber face and the acceptance angle of the fiber. If the reflected light has a Lambertian profile, then $\eta_{c,L}$ is equal to^29^

$$\eta_{c,L}=\sin\;\theta_{\mathrm{acc}}^{2}.$$ (11)

Since it is not known what the angular profile is for SFR measurements, we account for a possible non-Lambertian profile of photons reaching the fiber by modeling the collection efficiency as the Lambertian collection efficiency multiplied by a constant,

$$\eta_{c}=\sin\;\theta_{\mathrm{acc}}^{2}\cdot a_{1}.$$ (12)

An important check of our model is whether the fit parameter $a_{1}$ accurately relates to the collection efficiency. Therefore, we determined the reflectance values for simulations with a high value of $\mu_{s}^{\prime }d$ (1000; $\mu_{s}^{\prime }=10^{5}\;{\mathrm{cm}}^{-1}$, d=100  μm) and compared these to the collection efficiency $\eta_{c}$. For high values of $\mu_{s}^{\prime }d$, the reflectance will be diffuse and, thus, $R_{\mathrm{SFR},\mathrm{sb}}$ will approach 0 and $R_{\mathrm{dif}}$ will approach 1. Therefore, the reflectance should equal to the collection efficiency for high values of $\mu_{s}^{\prime }d$.

#### 2.2.2 Semiballistic component

For the semiballistic contribution to the reflectance, we searched for a parameter that would optimally capture the influence of the phase function. Usually, such attempts are based on the development of the phase function in Legendre polynomials:

$$p(\cos\;\theta )=\frac{1}{4\pi }\underset{n}{\sum }(2n+1)g_{n}P_{n}(\cos\;\theta ),$$ (13)

where p is the probability of scattering at an angle θ, $P_{n}$ are the Legendre polynomials of order N, and $g_{n}$ are the Legendre moments. The scattering model from Kanick et al.^7^ describes the influence of the phase function by incorporating the parameter $\gamma =(1-g_{2})/(1-g_{1})$. Next to γ, three other parameters have been proposed that could be used to incorporate the phase function influence in models to describe subdiffuse reflectance: δ ,^22^ σ,^23^ and $R_{p\mathrm{NA}}$.^9^ The parameters δ and σ also incorporate moments of the phase function: $\delta =(1-g_{3})/(1-g_{1})$ and $\sigma =\overset{\infty }{\underset{i=2}{\sum }}{\left(-0.5\right)}^{i-2}\frac{1-g_{i}}{1-g_{1}}$.

We recently introduced the parameter $R_{p\mathrm{NA}}$,^9^ to describe the semiballistic contribution to the reflectance for SFR spectroscopy. Photons were considered semiballistic if they had experienced a single backscatter event in combination with an arbitrary number of forward-scattering events upon detection. For these semiballistic photons, it was assumed that they would only be detected if all scattering events occurred at $\text{scattering angles}\leq \theta_{\mathrm{acc}}$. The full derivation is described in our previous paper.^9^ In short, based on this assumption, $R_{p\mathrm{NA}}$ is defined as

$$R_{p\mathrm{NA}}=\frac{p_{\mathrm{NA},b}}{1-p_{\mathrm{NA},f}}.$$ (14)

Here, $p_{\mathrm{NA},b}$ is the probability of a scattering event to occur within $\theta_{\mathrm{acc}}$ in the backward direction, which equals the integral of the phase function over the acceptance angle in the backward direction:

$$p_{\mathrm{NA},b}=2\pi \overset{\pi }{\underset{\pi -\theta_{\mathrm{acc}}}{\int }}p(\theta )\sin(\theta )d\theta .$$ (15)

Here $p_{\mathrm{NA},f}$ is the probability of a scattering event to occur within $\theta_{\mathrm{acc}}$ in the forward direction:

$$p_{\mathrm{NA},f}=2\pi \overset{\theta_{\mathrm{acc}}}{\underset{0}{\int }}p(\theta )\sin(\theta )d\theta .$$ (16)

We showed previously that for SFR spectroscopy the parameter $R_{p\mathrm{NA}}$ predicts the reflectance better than γ or σ,^9^ but we did not yet validate the assumptions behind the derivation of $R_{p\mathrm{NA}}$.

To test these assumptions here, we investigated the scattering angles of semiballistic photons that are detected. To this end, we examined the scattering angles of detected photons for the simulations with $\mu_{s}^{\prime }d=0.1$ and NAs of 0.10, 0.22, and 0.50. We extracted the normalized frequency distribution of scattering angles of detected photons from the MC simulations. We call this the “effective phase function.” Based on the results of the effective phase functions, we searched for a parameter that could better describe the influence of the phase function on the measured reflectance, by investigating the optimal integration limits for a parameter similar to $R_{p\mathrm{NA}}$, which we named $R_{p(\theta b,\theta f)}$:

$$R_{p(\theta b,\theta f)}=\frac{p_{b}(\theta_{b})}{1-p_{f}(\theta_{f})},$$ (17)

$$p_{b}(\theta_{b})=2\pi \overset{\pi }{\underset{\pi -\theta_{b}}{\int }}p(\theta )\sin(\theta )d\theta ,$$ (18)

$$p_{f}(\theta_{f})=2\pi \overset{\theta_{f}}{\underset{0}{\int }}p(\theta )\sin(\theta )d\theta .$$ (19)

**Fig. 1:**
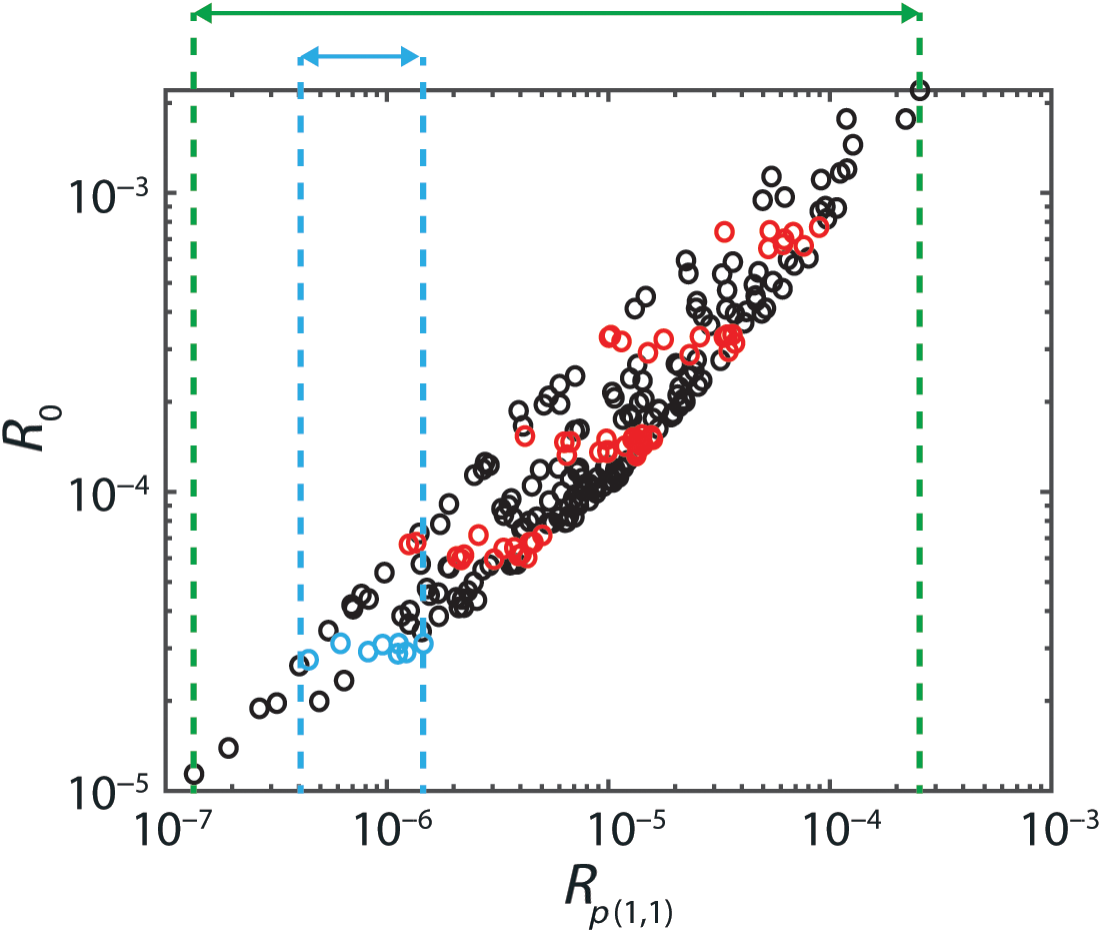
Concept of judging the suitability of the parameter $R_{p(\theta b,\theta f)}$ for a specific forward and backward integration angle. For a certain reflectance value ($R_{0}$), the dispersion of $R_{p(\theta b,\theta f)}$ for that reflectance (light blue) is divided by the entire range of $R_{p(\theta b,\theta f)}$ (green). This relative dispersion was determined for five reflectance values (depicted in red) for $\mu_{s}^{\prime }d=0.1$, per set of 207 simulations with the same NA.

We investigated the relation between $R_{0}$ and $R_{p(\theta b,\theta f)}$ for all possible backward and forward integration angles (in steps of 1 deg). Optimal integration angles were chosen based on a minimization of the dispersion in the parameter $\log\;10[R_{p(\theta b,\theta f)}]$ for a chosen reflectance, relative to the total range of $\log\;10[R_{p(\theta b,\theta f)}]$ (Fig. 1). For each NA, we determined the relative dispersion for five different reflectance values (±10%) equally spaced on a logarithmic scale. This relative dispersion is a measure for the suitability of $R_{p(\theta b,\theta f)}$ to model the reflectance, because the dispersion in the parameter will influence the uncertainty in all the estimated optical properties when $R_{p(\theta b,\theta f)}$ is used to model the reflectance. A relative measure is used because redefining the parameter by multiplying or subtracting a value would otherwise influence the measure.

Based on these results, we defined the parameter $p_{\mathrm{sb}}$ as

$$p_{\mathrm{sb}}=\frac{p_{b}(1\;\deg)}{1-p_{f}(23\;\deg)}.$$ (20)

We compared $p_{\mathrm{sb}}$ to γ, σ, δ, and $R_{p\mathrm{NA}}$ by determining the relative dispersion for three different reflectance values (±10%) per NA. Since we developed $p_{\mathrm{sb}}$ for the semiballistic contribution to the reflectance, we compared these parameters for $\mu_{s}^{\prime }d=0.1$, where most detected photons are semiballistic.

#### 2.2.3 Ratio diffuse and semiballistic photons

We use $p_{\mathrm{sb}}$ in the model for the ratio between semiballistic and diffuse photons X. For low values of $\mu_{s}^{\prime }d$, the diffuse reflectance scales with ${\left(\mu_{s}^{\prime }d\right)}^{2}$. Therefore, we include that factor in the denominator of Eq. (21). The semiballistic reflectance will scale differently with $\mu_{s}^{\prime }d$. We assume $R_{\mathrm{SFR},\mathrm{sb}}$ scales with $p_{\mathrm{sb}}{\left(\mu_{s}^{\prime }d\right)}^{-B}$. This difference in scaling is accounted for through the parameter $a_{3}$.

$$X=a_{2}{\left[\frac{p_{sb}}{{\left(\mu_{s}^{\prime }d\right)}^{2}}\right]}^{a_{3}}.$$ (21)

The parameters $a_{1}-a_{3}$ were determined by fitting the model [Eqs. (5)–(10), (12), (20)–(21)] to the MC results.

### 2.3 Monte Carlo Simulations Single Fiber Reflectance Spectra

To demonstrate the use of our model to extract optical properties from measured spectra, we performed MC simulations for Intralipid 20% diluted to a volume fraction of 0.05, using the scattering coefficient and phase function from literature^30^ and no absorption. To extract optical properties from SFR measurements, measurements with two fibers of different diameters are generally used (also referred to as multidiameter SFR or MDSFR). For robust fit results, the number of fit parameters should be considerably smaller than the number of data points. For an SFR measurement, the number of data points (reflectance values) is equal to the number of wavelengths, $\lambda_{n}$. Separate values for $\mu_{s}^{\prime }$ and $p_{\mathrm{sb}}$ per wavelength cannot be determined directly using a fit on an SFR spectrum, since the number of fit parameters would equal $2\lambda_{n}$. Therefore, the number of fit parameters is reduced by modeling the reduced scattering coefficient as $\mu_{s}^{\prime }=a\cdot {\left(\lambda /\lambda_{0}\right)}^{-b}$, where a is the scattering amplitude, b is the scattering slope, and $\lambda_{0}$ is a reference wavelength. The wavelength dependence of tissue phase functions, in general, and $p_{\mathrm{sb}}$, specifically, is not well characterized. Therefore, a value of $p_{\mathrm{sb}}$ will have to be fitted for each wavelength within the spectrum. For a single measured spectrum, the number of data points will equal $\lambda_{n}$, but the number of fit parameters will equal $2+\lambda_{n}$ (2 for $\mu_{s}^{\prime }$ and $\lambda_{n}$ for $p_{\mathrm{sb}}$). Such an underdetermined system will not provide robust fit results. To overcome this issue, measurements can be performed using two different fiber diameters. In that case, the number of data points will equal $2\lambda_{n}$ and the number of fit parameters will equal $2+\lambda_{n}$. Therefore, we performed MC simulations of spectra for two different fiber diameters: 300 and 600  μm. We modeled spectra from 400 to 900 nm in steps of 5 nm. For the fit of $\mu_{s}^{\prime }$, we used a reference wavelength of 600 nm.

## 3 Results

### 3.1 Phase Function Parameter Semiballistic Contribution ($p_{\mathrm{sb}}$)

For $\mu_{s}^{\prime }d=0.1$ and NAs of 0.10, 0.22, and 0.50, the contribution of semiballistic photons (with only a single backscattering event and any number of forward-scattering events) to the total reflectance was 99%, 98%, and 98%, respectively. Therefore, we used these simulations to examine the semiballistic contribution to the reflectance.

**Fig. 2:**
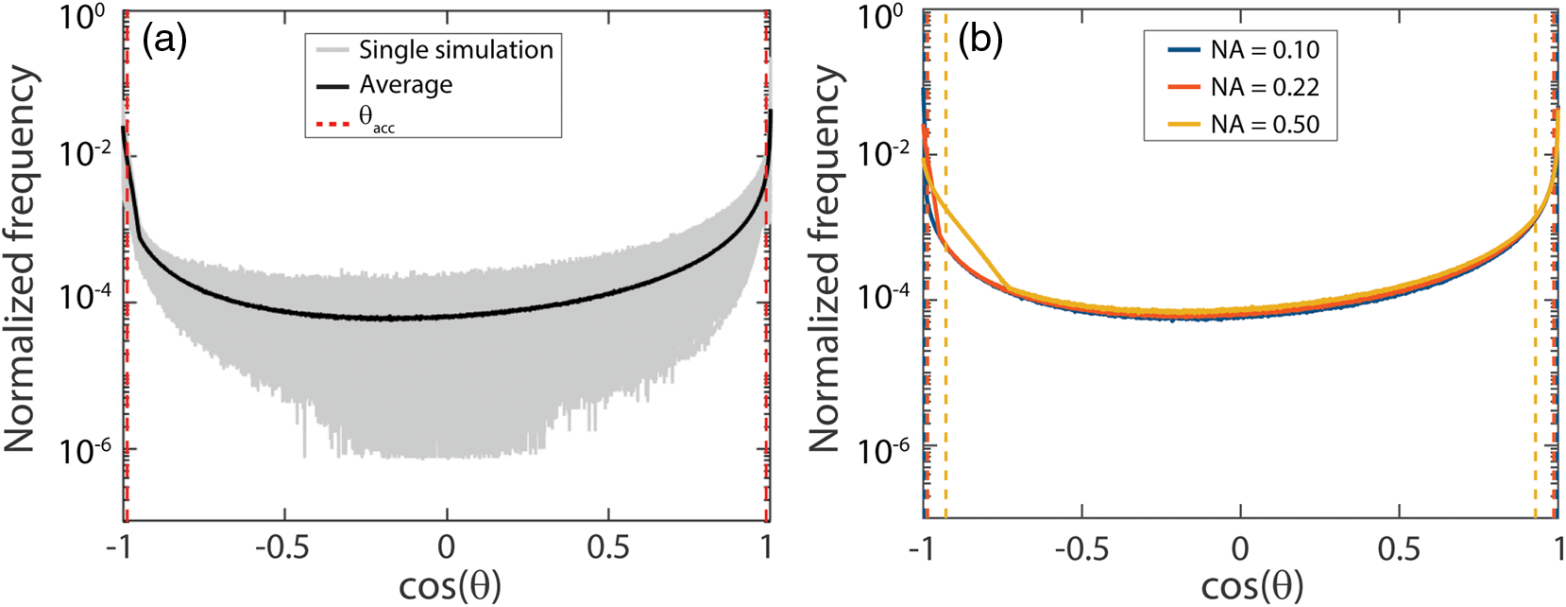
(a) Effective phase function: frequency distribution of scattering angles of detected photons for all 207 simulations with $\mu_{s}^{\prime }d=0.1$ ($\mu_{s}^{\prime }=10\;{\mathrm{cm}}^{-1}$, d=100  μm) and NA=0.22. For each simulation, the frequency distribution is normalized to 1. The gray lines represent individual effective phase functions, the black line indicates the average effective phase function, and the red dashed lines indicate the acceptance angle of the fiber, $\theta_{\mathrm{acc}}$. (b) Average effective phase functions for $\mu_{s}^{\prime }d=0.1$ ($\mu_{s}^{\prime }=10\;{\mathrm{cm}}^{-1}$, d=100  μm) and NA=0.10, 0.22, or 0.50. The dashed lines indicate $\theta_{\mathrm{acc}}$ for each NA. Detected photons also underwent scattering angles outside $\theta_{\mathrm{acc}}$.

To investigate the assumption behind $R_{p\mathrm{NA}}$ that semiballistic photons are only detected if all the scattering events occurred at $\text{scattering angles}\leq \theta_{\mathrm{acc}}$, we determined the effective phase functions (the frequency distributions of the scattering angles of detected photons) for each simulation. From these effective phase functions, it can be concluded that detected photons also underwent scattering angles outside $\theta_{\mathrm{acc}}$ (Fig. 2). As depicted in Fig. 2(b), the NA has almost no influence on the effective phase function for angles in the forward direction. The NA does have an influence on the percentage of detected scattering events for angles in the backward direction. The sharp increase corresponds to twice $\theta_{\mathrm{acc}}$, which can be explained by the fact that photons are launched with a random angle within the acceptance angle and that photons are only detected when they arrive at the fiber with an angle within the acceptance angle of the fiber. A photon that is launched at $\theta_{\mathrm{acc}}$ and undergoes only a single scattering event can be detected if that scattering angle is $2\cdot \theta_{\mathrm{acc}}$.

**Fig. 3:**
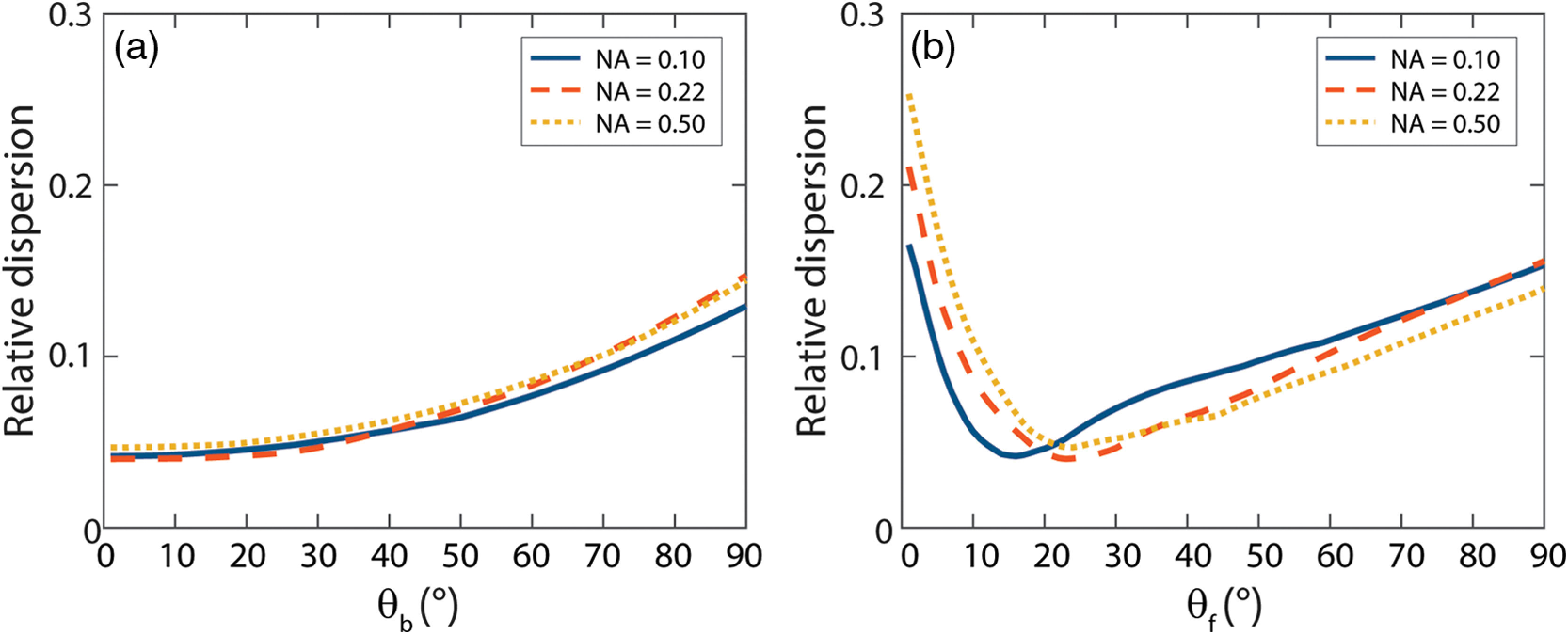
Relative dispersion versus integration angle for simulations with NAs of 0.10, 0.22, and 0.50, $\mu_{s}^{\prime }d=0.1$ (a) Relative dispersion versus backward integration angle, for the optimal forward integration angles (16 deg, 23 deg, and 23 deg, respectively). (b) Relative dispersion versus forward integration angle, for the optimal backward integration angle (1 deg for all three NAs). The relative dispersion is much more sensitive to the forward integration angle than the backward integration angle and is slightly influenced by the NA.

Since detected photons also underwent scattering events at angles larger than $\theta_{\mathrm{acc}}$, we searched for a parameter that could better describe the influence of the phase function on the measured reflectance, by investigating the optimal integration limits for $R_{p(\theta b,\theta f)}$ [Eqs. (17)–(19)]. The optimization of the integration angles was based on minimizing the relative dispersion in the parameter $\log\;10[R_{p(\theta b,\theta f)}]$. Figure 3(a) depicts the relative dispersion versus backward integration angle, for the optimal forward integration angle per NA. The relative dispersion is fairly constant for integration angles up to 20 deg and the results are similar for different NAs. Figure 3(b) depicts the relative dispersion versus forward integration angle, for the optimal backward integration angle per NA. There is a sharp optimum for the forward integration angle and the optimum integration angle varies with the NA. For each NA, the optimal integration angles can be found in Table 2.

**Table 2:** Optimal integration angles backward ($\theta_{b}$) and forward ($\theta_{f}$) for different values of the NA.

NA	$\theta_{b}$	$\theta_{f}$
0.10	1	16
0.22	1	23
0.50	1	23

Since fibers with an NA of 0.22 are generally used for SFR measurements, we optimized our parameter for this NA. The optimal integration angle that results in the lowest relative dispersion is 1 deg backward and 23 deg forward. Therefore, we will use $p_{\mathrm{sb}}$ [Eq. (20)] in our model for the semiballistic contribution to the reflectance.

**Fig. 4:**
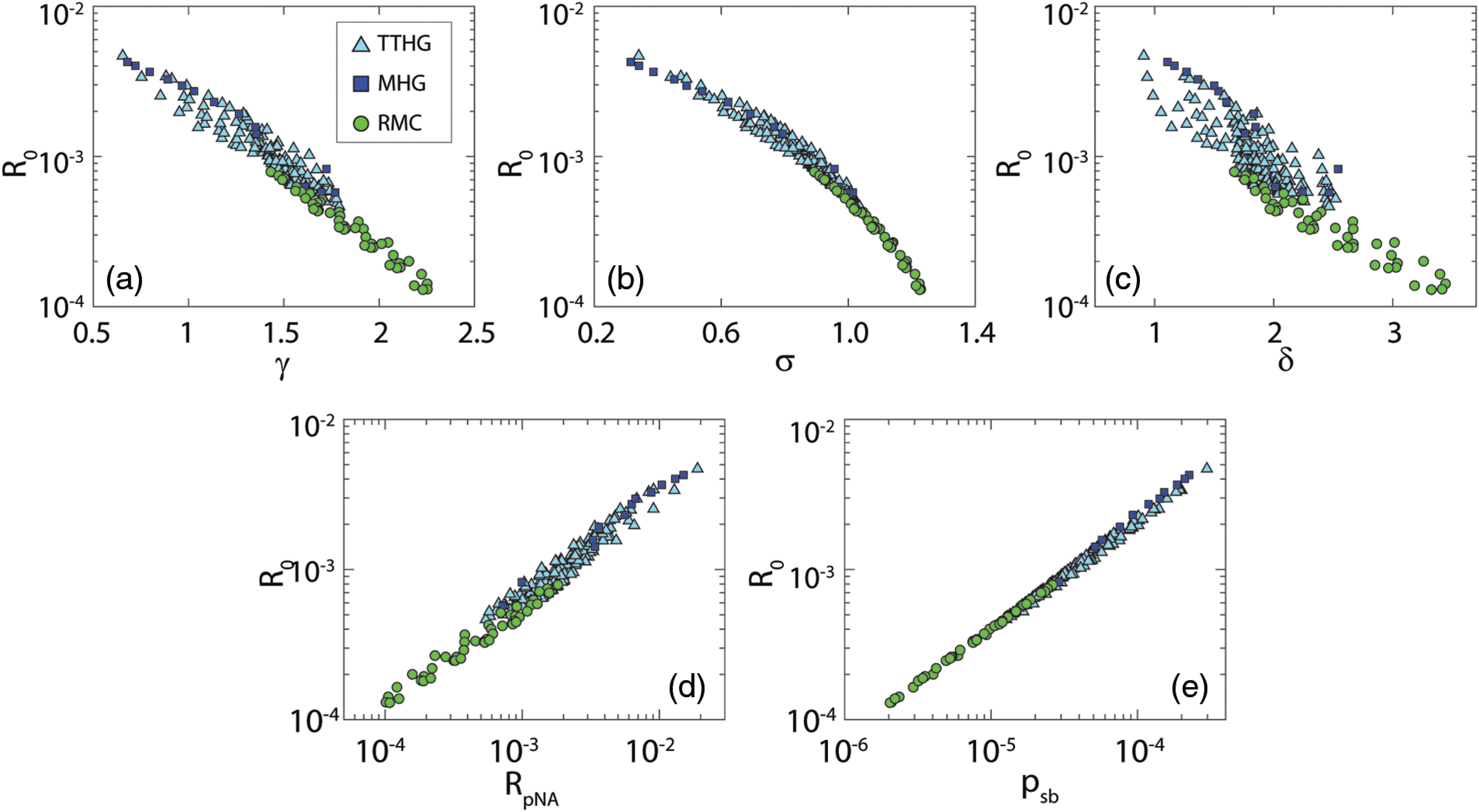
Simulated reflectance ($R_{0}$) versus γ, σ, δ, $R_{p\mathrm{NA}}$, and $p_{\mathrm{sb}}$, for an NA of 0.22 and $\mu_{s}^{\prime }d=0.1$; colors indicate phase function types. Note the log scales for both the reflectance, $R_{p\mathrm{NA}}$, and $p_{\mathrm{sb}}$. Here $p_{\mathrm{sb}}$ is most closely related to the semiballistic reflectance.

Figure 4 shows the relation between the reflectance and γ, σ, δ, $R_{p\mathrm{NA}}$, and $p_{\mathrm{sb}}$ for simulations with an NA of 0.22 and $\mu_{s}^{\prime }d=0.1$. Overall, $p_{\mathrm{sb}}$ has a lower relative dispersion. A quantitative measure of the dispersion was calculated by determining the relative dispersion of each parameter for three reflectance values per NA (Table 3). For an NA of 0.10, $p_{\mathrm{sb}}$ outperforms the other parameters for all three reflectance values. For an NA of 0.22, $p_{\mathrm{sb}}$ outperforms the other parameters for the lower three reflectance values but has a higher relative dispersion for the highest reflectance value. For an NA of 0.50, the results for $R_{p\mathrm{NA}}$ and $p_{\mathrm{sb}}$ are similar (and both outperform the other parameters), which is expected since the integration angles for $R_{p\mathrm{NA}}$ are 22 deg for an NA of 0.50.

**Table 3:** Variability of $p_{\mathrm{sb}}$, $R_{p\mathrm{NA}}$, σ, δ, and γ for $\mu_{s}^{\prime }d=0.1$, defined as the dispersion in $p_{\mathrm{sb}}$, $R_{p\mathrm{NA}}$, σ, and γ values for a chosen reflectance (+/- 10%) relative to the total range of each parameter.

NA	Reflectance	Variability				
		$p_{\mathrm{sb}}$	$R_{p\mathrm{NA}}$	σ	δ	γ
0.10	0.0001	0.013	0.030	0.042	0.178	0.074
	0.0002	0.045	0.104	0.092	0.344	0.183
	0.0004	0.087	0.153	0.138	0.322	0.226
0.22	0.0005	0.016	0.029	0.050	0.231	0.111
	0.001	0.045	0.060	0.114	0.272	0.185
	0.003	0.217	0.130	0.096	0.095	0.086
0.50	0.001	0.002	0.001	0.015	0.131	0.056
	0.003	0.030	0.035	0.089	0.344	0.185
	0.005	0.088	0.115	0.141	0.322	0.226

### 3.2 Scattering Model

Table 4 lists the fit parameters obtained from fitting Eqs. (5)–(10), (12), and (20)–(21) to all the MC simulations. For all three NAs, the value of $a_{1}$ is similar. Therefore, we chose a single value for $a_{1}$ for all three NAs and repeated the fit to obtain values for $a_{2}$ and $a_{3}$ for each NA. This resulted in a new set of parameter values to be used in the SFR scattering model (Table 5).

**Table 4:** Resulting fit parameters per NA using Eqs. (5)–(10), (12), (20), and (21) on simulated SFR measurements. The 95% confidence intervals on these fit parameters are indicated.

	NA=0.10		NA=0.22		NA=0.50	
	Value	95% CI	Value	95% CI	Value	95% CI
$a_{1}$	1.130	(±0.006)	1.119	(±0.005)	1.098	(±0.005)
$a_{2}$	4427	(±87)	3065	(±50)	1461	(±22)
$a_{3}$	0.785	(±0.003)	0.750	(±0.003)	0.684	(±0.002)

**Table 5:** Resulting fit parameters per NA using Eqs. (5)–(10), (12), (20), and (21) and $a_{1}=1.11$ on simulated SFR measurements. The 95% confidence intervals on these fit parameters are indicated.

	NA=0.10		NA=0.22		NA=0.50	
	Value	95% CI	Value	95% CI	Value	95% CI
$a_{2}$	4370	(±87)	3046	(±49)	1475	(±23)
$a_{3}$	0.780	(±0.003)	0.748	(±0.003)	0.688	(±0.003)

To determine whether the fit parameter $a_{1}$ accurately relates to the collection efficiency, we compared the reflectance values for simulations with a high value of $\mu_{s}^{\prime }d$ (1000; $\mu_{s}^{\prime }=10^{5}\;{\mathrm{cm}}^{-1}$, d=0.01  cm) to the collection efficiency using Eq. (12) in combination with the $a_{1}$ values from Table 4. For high values of $\mu_{s}^{\prime }$, the reflectance should equal the collection efficiency. The differences are 1.1%, 1.6%, and 0.7% for NAs of 0.10, 0.22, and 0.50, respectively.

**Fig. 5:**
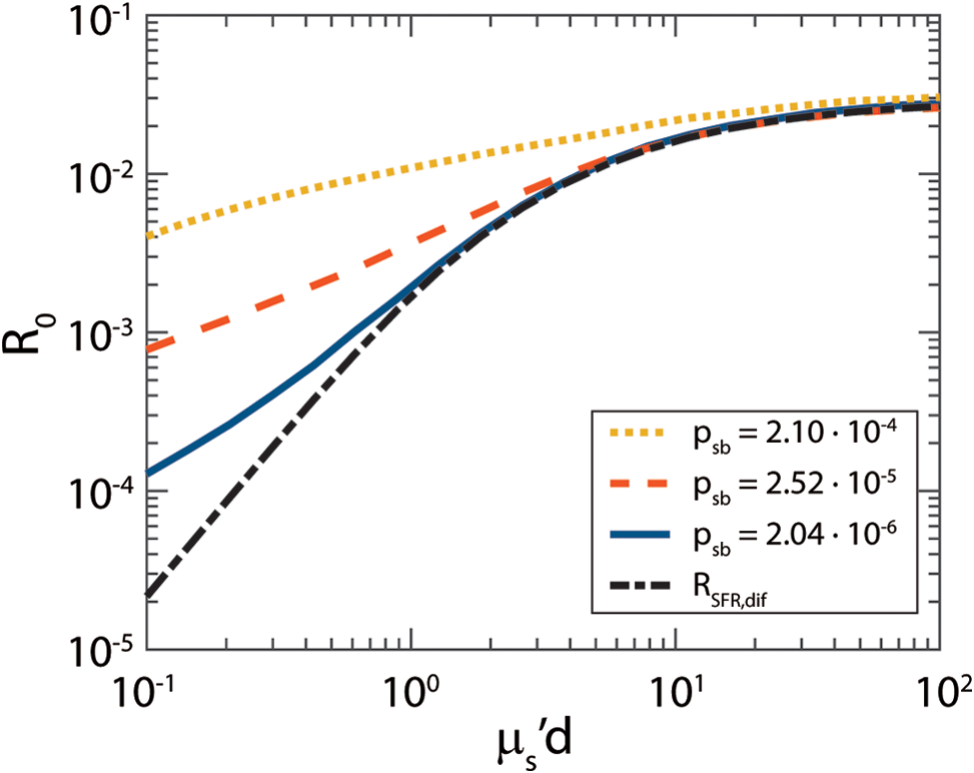
Simulated $R_{0}$ as a function of $\mu_{s}^{\prime }d$. For lower values of $\mu_{s}^{\prime }d$, an additional semiballistic reflectance is added to the diffuse reflectance ($R_{\mathrm{SFR},dif}$). With increasing $p_{\mathrm{sb}}$, the fraction of semiballistic photons increases.

Using the parameter $a_{1}$, we can now calculate the diffuse contribution to the reflectance ($R_{\mathrm{SFR},\mathrm{dif}}$) and show that the reflectance ($R_{0}$) is indeed a sum of a diffuse and a semiballistic component (Fig. 5). For high values of $\mu_{s}^{\prime }d$, the total reflectance equals the diffuse reflectance (dashed black line), but for lower values of $\mu_{s}^{\prime }d$ there is an additional semiballistic contribution to the reflectance. With increasing $p_{\mathrm{sb}}$, the fraction of semiballistic photons increases.

### 3.3 Accuracy Scattering Model

**Fig. 6:**
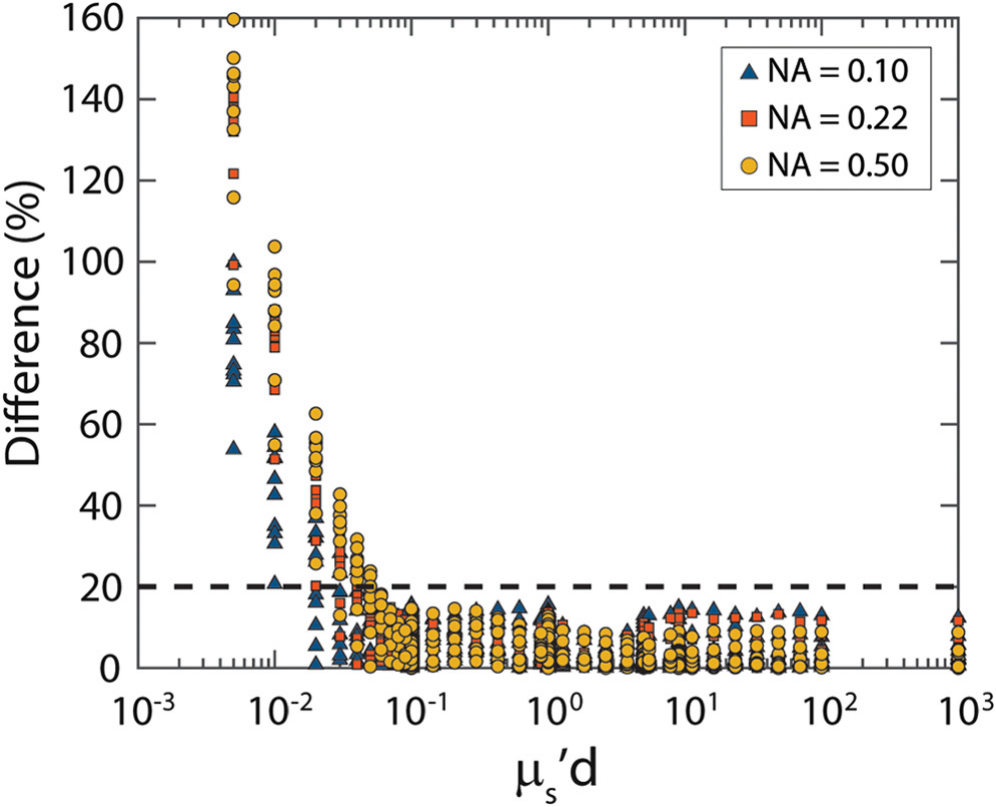
Absolute value of the difference (%) between the model and the MC simulations versus $\mu_{s}^{\prime }d$. For low values of $\mu_{s}^{\prime }d$, the difference increases significantly.

To determine the accuracy of the scattering model, we used Eqs. (5)–(10), (12), and (20)–(21), in combination with the parameters from Table 5, to determine the difference between the reflectance predicted by the model to the reflectance from the MC simulations. First, we investigated the limit with respect to $\mu_{s}^{\prime }d$ for our model, by determining the difference for all the simulations with 10 different phase functions ($0.005<\mu_{s}^{\prime }d<10^{3}$). For values of $\mu_{s}^{\prime }d$ that are lower than 0.1, the difference increases significantly (Fig. 6).

**Fig. 7:**
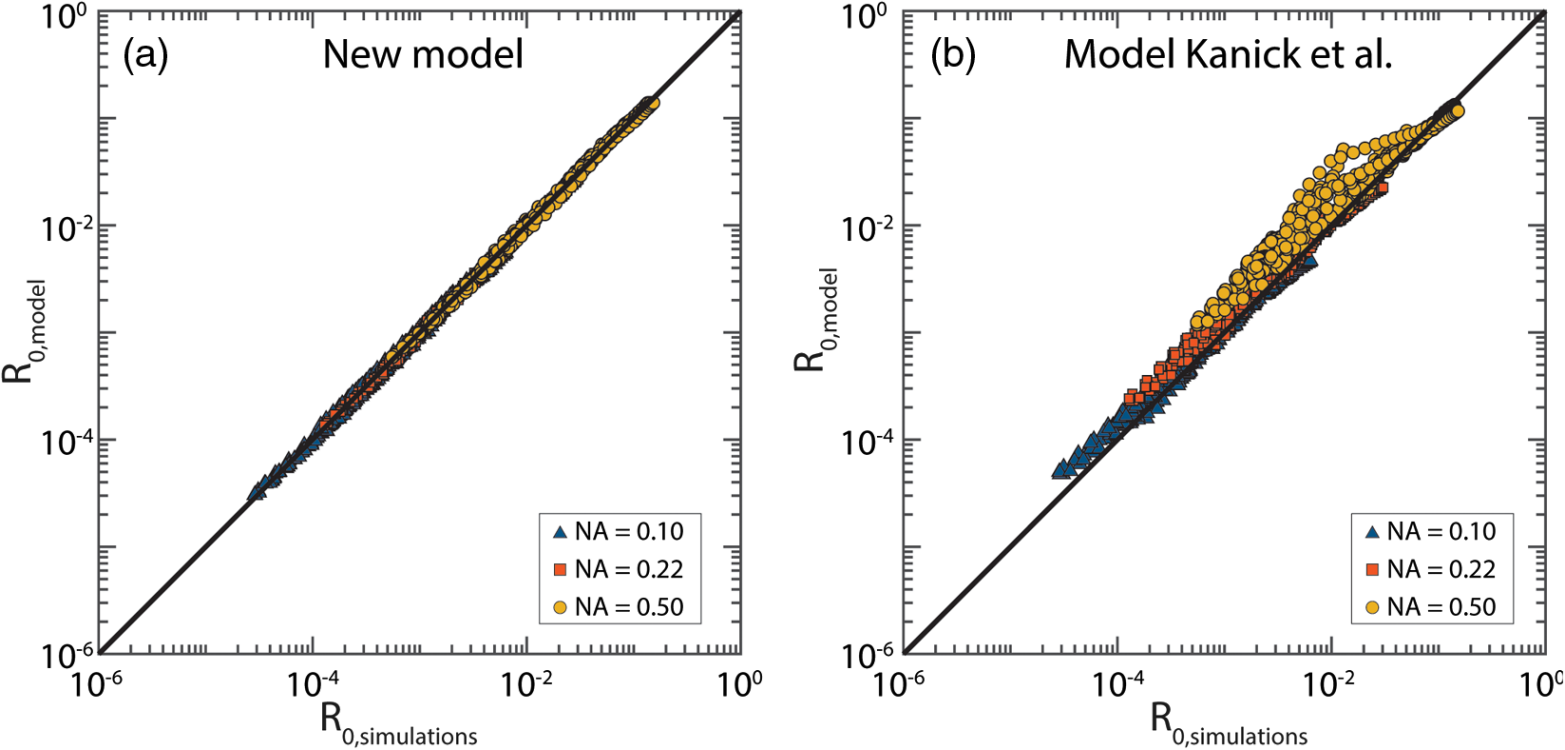
Reflectance as predicted by the model ($R_{0,\text{model}}$) versus the reflectance obtained from the MC simulations ($R_{0,\text{simulations}}$). The black line depicts the perfect prediction. (a) For our model, the median error is 2.1% with a standard deviation of 3.0%. (b) For the model from Kanick et al.,^7^ the median error is 9.0% with a standard deviation of 36.8%.

Next, we determined the difference between the reflectance predicted by the model and the reflectance from the MC simulations for all simulations where $\mu_{s}^{\prime }d\geq 0.1$ (Fig. 7). The median error is 2.1%, with a standard deviation of 3.0% and a maximum error of 16%. The error in the predicted reflectance is similar for all three NA values. The errors are much lower compared to the model from Kanick et al.^7^ [Fig. 7(b)], where the median error is 9.0%, with a standard deviation of 36.8% and a maximum error of 303%.

### 3.4 Monte Carlo Simulations Single Fiber Reflectance Spectra

**Fig. 8:**
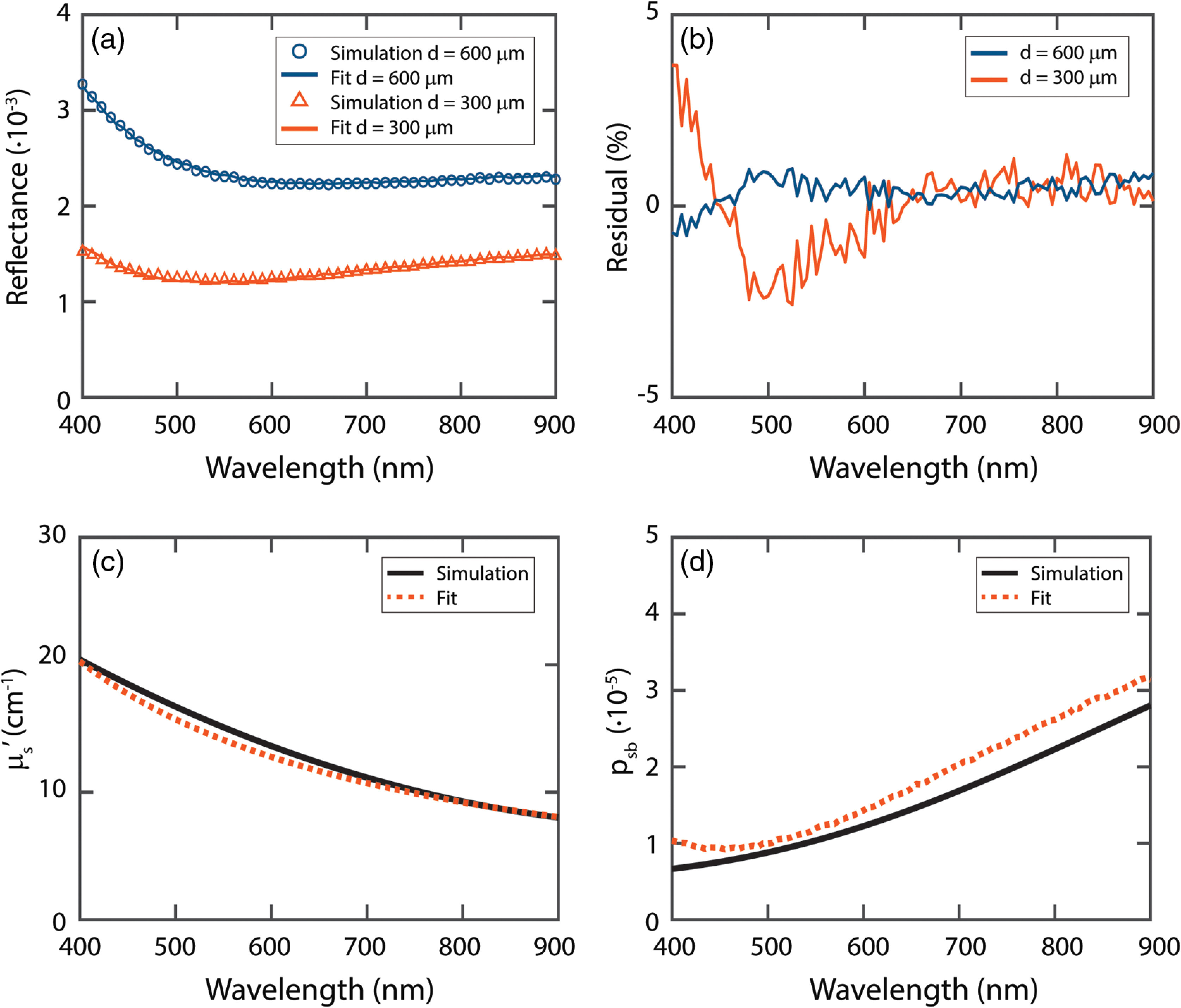
(a) Fit on simulated spectra for Intralipid 20%, diluted to a volume fraction of 0.05. (b) Residual of fit. (c) Fit result for the reduced scattering coefficient. (d) Fit result for $p_{\mathrm{sb}}$.

Figure 8 shows the simulated spectra for Intralipid 20%, diluted to a volume fraction of 0.05. The residual of the fit is below 5% for the 300-μm fiber and fluctuates around 0% for the 600-μm fiber. The obtained $\mu_{s}^{\prime }$ and $p_{\mathrm{sb}}$ values are close to the values used in the simulations.

## 4 Discussion

SFR spectroscopy is a technique that has a sampling depth of a few hundred micrometers and has, therefore, the potential to detect local, small-scale changes in tissue—such as small or superficial tumors. However, the model currently used to obtain optical scattering properties from SFR measurements is limited to tissues with MHG phase functions. Here, we developed a new model that accurately relates the reflectance in the absence of absorption ($R_{0}$) to scattering properties of tissue for a wide range of phase functions, scattering coefficients, fiber diameters, and NAs. Our model predicts $R_{0}$ substantially better compared to the model from Kanick et al.^7^ (2.1% versus 9.0% median error). To determine optical properties of tissues with SFR spectroscopy, the next step will be to add absorption to the model.

We demonstrated the use of our model to extract optical properties from SFR spectra, using simulated spectra for two fibers of different diameters (MDSFR). Since there is currently no model available for the wavelength dependence of $p_{\mathrm{sb}}$, a measurement with two fibers of different diameters is required. The fitted values we obtained for $\mu_{s}^{\prime }$ are very close to the simulated $\mu_{s}^{\prime }$. The difference between the fitted and the simulated values of $p_{\mathrm{sb}}$ are slightly larger. A model for the wavelength-dependent behavior of $p_{\mathrm{sb}}$ could further simplify the measurements, since it would enable the use of only a single fiber.

We model $R_{0}$ as a sum of a diffuse and a semiballistic contribution. For the semiballistic contribution to the reflectance, we proposed a new parameter, $p_{\mathrm{sb}}$, to capture the influence of the phase function. Our findings imply that the semiballistic contribution is mainly influenced by the probability of photons scattering forward within 23 deg. A physical reason for this angular limit is yet to be found. The suitability of $R_{p(\theta b,\theta f)}$ to model the semiballistic contribution depends much more on the forward integration angle than the backward integration angle, even though this might not be expected based on our results for the effective phase functions. The large influence of the forward integration angle might be explained by the fact that semiballistic photons undergo only a single backscatter event, but generally undergo multiple forward-scattering events. Even though we had chosen $p_{\mathrm{sb}}$ to optimize the parameter for an NA of 0.22, the model performed similarly well for all three NAs. Previous work on subdiffuse scattering presented the subdiffuse parameters γ, δ, σ, and $R_{p\mathrm{NA}}$. The parameters γ, δ, and σ are all based on the Legendre moments of the phase function. It can be concluded that the Legendre moments are not the most suitable for describing the semiballistic contribution to SFR measurements.

We model the diffuse contribution to the reflectance by solving the model for spatially resolved reflectance of Farrell et al.^17^ for an overlapping source and detection fiber.^18^ For low values of $\mu_{s}^{\prime }d$, the diffuse reflectance scales with ${\left(\mu_{s}^{\prime }d\right)}^{2}$, which justifies the inclusion of that factor in the denominator of the ratio between semiballistic and diffuse photons. The semiballistic reflectance scaled differently with $\mu_{s}^{\prime }d$, which is accounted for in the parameter $a_{3}$.

As expected, the fraction of diffuse photons increases for higher values of $\mu_{s}^{\prime }d$ and lower values of $p_{\mathrm{sb}}$. Thus, the fiber diameter will influence the ratio of semiballistic to diffuse photons that are detected.

In our model, we model the collection efficiency as ∼1.11 times the collection efficiency that would be expected if the light that reached the fiber face would have had a Lambertian profile. We determined that this indeed still represents the collection efficiency by showing that, for very high values of $\mu_{s}^{\prime }d$, the difference between the collection efficiency and the reflectance was around 1%. Another assumption for the collection efficiency could be that light reaching the fiber face has a flat angular distribution. For an NA of 0.22, this would result in a collection efficiency of 3.8%, compared to 2.7% for a Lambertian profile.^31^ In our model, we use 3% for this NA. This suggests that the angular profile of light reaching the fiber face is in between a Lambertian and a flat angular distribution.

Since part of our model is based on diffusion theory, it can be expected that there is a limit where $\mu_{s}^{\prime }d$ is too low for diffusion theory to accurately describe the diffuse contribution to the reflectance. For values of $\mu_{s}^{\prime }d$ below 0.1, our model becomes inaccurate. For tissues with low values of $\mu_{s}^{\prime }$, it is, therefore, advisable to use fibers with a larger diameter. For example, with a 500-μm fiber, our model accurately predicts the reflectance for $\mu_{s}^{\prime }\geq 2\;{\mathrm{cm}}^{-1}$, and for most tissues $\mu_{s}^{\prime }$ will be larger than $2\;{\mathrm{cm}}^{-1}$.^32^

For the derivation of our model, we have included a range of phase functions that have been measured in tissue. As far as we know, no other tissue phase functions have been measured yet, but new phase functions might be discovered in the future. In that case, it will have to be investigated whether the model is still accurate. Nevertheless, the tested phase functions are widely variable in shape, most importantly within the forward and backward integration limits of the phase function.

Our findings could have implications for other subdiffuse techniques. For other techniques, such as spatially resolved reflectance, it will be interesting to see whether $R_{p(\theta b,\theta f)}$ can also be used to model the semiballistic contribution to the reflectance and, if so, which integration angles will have to be used.

Furthermore, since semiballistic photons have shorter pathlengths than diffuse photons, time-resolved or low coherence-based subdiffuse techniques could be developed to separate the semiballistic and diffuse contributions to the reflectance.^33^ Ultimately, this would lead to a understanding of the pathlength distribution (l=ct), and possible application of Eq. (1) to derive models for SFR spectroscopy.

## 5 Conclusion

We developed a model for the reflectance in the absence of absorption ($R_{0}$) measured with SFR spectroscopy that provides accurate results for a wide range of tissue phase functions. Since the phase function of a specific tissue under investigation is generally not known, a model that is valid for the wide range of phase functions that can be encountered in tissue is essential. We modeled the reflectance as the sum of a diffuse and a semiballistic component. We used the model of Farrell et al.^17^ for the diffuse component, solved for an overlapping source and detector fiber. We proposed the parameter $p_{\mathrm{sb}}$ to model the influence of the phase function on the semiballistic contribution to the reflectance. For SFR measurements, $p_{\mathrm{sb}}$ outperforms the subdiffuse parameters γ, δ, σ, and $R_{p\mathrm{NA}}$. The new model predicts the measured reflectance substantially better, compared to the commonly used model from Kanick et al.^7^ To determine the tissue optical properties accurately with SFR spectroscopy, the next step will be to include absorption in the model.

## References

[1] Hariri Tabrizi S. et al., “Single fiber reflectance spectroscopy on cervical premalignancies: the potential for reduction of the number of unnecessary biopsies,” J. Biomed. Opt. 18(1), 017002 (2013). JBOPFO 1083-3668 10.1117/1.JBO.18.1.017002 23292613

[r2] Stegehuis P. L. et al., “Toward optical guidance during endoscopic ultrasound-guided fine needle aspirations of pancreatic masses using single fiber reflectance spectroscopy: a feasibility study,” J. Biomed. Opt. 22(2), 024001 (2017). JBOPFO 1083-3668 10.1117/1.JBO.22.2.024001 28170030

[r3] Sircan-Kuçuksayan A. Denkceken T. Canpolat M., “Differentiating cancerous tissues from noncancerous tissues using single-fiber reflectance spectroscopy with different fiber diameters,” J. Biomed. Opt. 20(11), 115007 (2015). JBOPFO 1083-3668 10.1117/1.JBO.20.11.115007 26590218

[r4] Gamm U. A. et al., “*In vivo* determination of scattering properties of healthy and malignant breast tissue by use of multi-diameter-single fiber reflectance spectroscopy (MDSFR),” Proc. SPIE 8592, 85920T (2013). 10.1117/12.2008852

[r5] Bugter O. et al., “Optical pre-screening for laryngeal cancer using reflectance spectroscopy of the buccal mucosa,” Biomed. Opt. Express 9(10), 4665 (2018). BOEICL 2156-7085 10.1364/BOE.9.004665 30319894 PMC6179391

[6] Bugter O. et al., “Optical detection of field cancerization in the buccal mucosa of patients with esophageal cancer,” Clin. Transl. Gastroenterol. 9(4), 152 (2018). 10.1038/s41424-018-0023-6 29712897 PMC5928160

[7] Kanick S. C. et al., “Method to quantitatively estimate wavelength-dependent scattering properties from multidiameter single fiber reflectance spectra measured in a turbid medium,” Opt Lett. 36(15), 2997–2999 (2011). 10.1364/OL.36.002997 21808384

[8] Kanick S. C. et al., “Monte Carlo analysis of single fiber reflectance spectroscopy: photon path length and sampling depth,” Phys. Med. Biol. 54, 6991–7008 (2009). PHMBA7 0031-9155 10.1088/0031-9155/54/22/016 19887712

[9] Post A. L. et al., “Modeling subdiffusive light scattering by incorporating the tissue phase function and detector numerical aperture,” J. Biomed. Opt. 22(5), 050501 (2017). JBOPFO 1083-3668 10.1117/1.JBO.22.5.050501 28530013

[10] Marchesini R. et al., “Extinction and absorption coefficients and scattering phase functions of human tissues *in vitro*,” Appl. Opt. 28(12), 2318 (1989). 10.1364/AO.28.002318 20555518

[11] Saccomandi P. et al., “Estimation of anisotropy coefficient of swine pancreas, liver and muscle at 1064 nm based on goniometric technique,” J. Biophotonics 8(5), 422–428 (2014). 10.1002/jbio.v8.5 24995557

[12] van der Zee P. Essenpreis M. Delpy D. T., “Optical properties of brain tissue,” Proc. SPIE 1888, 454–465 (1993). PSISDG 0277-786X 10.1117/12.154665

[13] Ghosh N. et al., “Measurement of optical transport properties of normal and malignant human breast tissue,” Appl. Opt. 40(1), 176–184 (2001). 10.1364/AO.40.000176 18356989

[14] Zijp J. ten Bosch J., “Optical properties of bovine muscle tissue *in vitro*; a comparison of methods,” Phys. Med. Biol. 43(10), 3065–3081 (1998). PHMBA7 0031-9155 10.1088/0031-9155/43/10/026 9814535

[15] Reynolds L. O. McCormick N. J., “Approximate two-parameter phase function for light scattering,” J. Opt. Soc. Am. 70(10), 1206–1212 (1980). JOSAAH 0030-3941 10.1364/JOSA.70.001206

[16] Yaroslavsky A. N. et al., “Optical properties of blood in the near-infrared spectral range,” Proc. SPIE 2678, 314–324 (1996). PSISDG 0277-786X 10.1117/12.239516

[17] Farrell T. J. Patterson M. S. Wilson B., “A diffusion theory model of spatially resolved, steady-state diffuse reflectance for the noninvasive determination of tissue optical properties *in vivo*,” Med. Phys. 19(4), 879–888 (1992). MPHYA6 0094-2405 10.1118/1.596777 1518476

[18] Faber D. J. et al., “Analytical model for diffuse reflectance in Single Fiber Reflectance Spectroscopy,” Opt. Lett. (2019). 10.1364/OL.385845 32236072

[19] Mourant J. R. et al., “Influence of the scattering phase function on light transport measurements in turbid media performed with small source-detector separations,” Opt. Lett. 21(7), 546–548 (1996). OPLEDP 0146-9592 10.1364/OL.21.000546 19865467

[20] Kienle A. Forster F. K. Hibst R., “Influence of the phase function on determination of the optical properties of biological tissue by spatially resolved reflectance,” Opt. Lett. 26(20), 1571–1573 (2001). OPLEDP 0146-9592 10.1364/OL.26.001571 18049666

[21] Bevilacqua F. Depeursinge C., “Monte Carlo study of diffuse reflectance at source-detector separations close to one transport mean free path,” J. Opt. Soc. Am. A 16(12), 2935 (1999). JOAOD6 0740-3232 10.1364/JOSAA.16.002935

[22] Naglič P. et al., “Adopting higher-order similarity relations for improved estimation of optical properties from subdiffusive reflectance,” Opt. Lett. 42(7), 1357–1360 (2017). OPLEDP 0146-9592 10.1364/OL.42.001357 28362768

[23] Bodenschatz N. et al., “Quantifying phase function influence in subdiffusively backscattered light,” J. Biomed. Opt. 21(3), 035002 (2016). JBOPFO 1083-3668 10.1117/1.JBO.21.3.035002 26968384

[24] Prahl S. A., “A Monte Carlo model of light propagation in tissue,” Proc. SPIE 10305(January), 1030509 (1989). PSISDG 0277-786X 10.1117/12.2283590

[25] Wang L. V. Jacques S. L. Zheng L., “MCML—Monte Carlo modeling of light transport in multi-layered tissues,” Comput. Methods Programs Biomed. 47, 131–146 (1995). CMPBEK 0169-2607 10.1016/0169-2607(95)01640-F 7587160

[26] Zijp J. R. ten Bosch J. J., “Use of tabulated cumulative density functions to generate pseudorandom numbers obeying specific distributions for Monte Carlo simulations,” Appl. Opt. 33(3), 533 (1994). APOPAI 0003-6935 10.1364/AO.33.000533 20862045

[27] Solomon H., Geometric Probability. Society for Industrial and Applied Mathematics, Philadelphia (1978).

[28] Martelli F. et al., “Photon migration through a turbid slab described by a model based on diffusion approximation. II. Comparison with Monte Carlo results,” Appl. Opt. 36(19), 4600 (1997). APOPAI 0003-6935 10.1364/AO.36.004600 18259255

[29] Bargo P. R. Prahl S. A. Jacques S. L., “Collection efficiency of a single optical fiber in turbid media,” Appl. Opt. 42(16), 3187–3197 (2003). APOPAI 0003-6935 10.1364/AO.42.003187 12790469

[30] Michels R. Foschum F. Kienle A., “Optical properties of fat emulsions,” Opt. Express 16(8), 5907–5925 (2008). OPEXFF 1094-4087 10.1364/OE.16.005907 18542702

[31] Martelli F. et al., “Properties of the light emerging from a diffusive medium: angular dependence and flux at the external boundary,” Phys. Med. Biol. 44(5), 1257–1275 (1999). PHMBA7 0031-9155 10.1088/0031-9155/44/5/013 10368017

[32] Jacques S. L., “Optical properties of biological tissues: a review,” Phys. Med. Biol. 58(14), 5007–5008 (2013). PHMBA7 0031-9155 10.1088/0031-9155/58/14/5007 23666068

[33] Petoukhova A. L. Steenbergen W. de Mul F. F. M., “Path-length distribution and path-length-resolved Doppler measurements of multiply scattered photons by use of low-coherence interferometry,” Opt. Lett. 26(19), 1492 (2001). OPLEDP 0146-9592 10.1364/OL.26.001492 18049645

